# Supplementary material for: Identification of Biomarkers for Methamphetamine Exposure Time Prediction in Mice Using Metabolomics and Machine Learning Approaches
Source: Metabolites. 2022 Dec 10;12(12):1250. doi: 10.3390/metabo12121250 (PMC9780981; doi:10.3390/metabo12121250)
Supplement: Supplementary file 1 [file metabolites-12-01250-s001.zip › Figure legends.pdf]

Figure legends

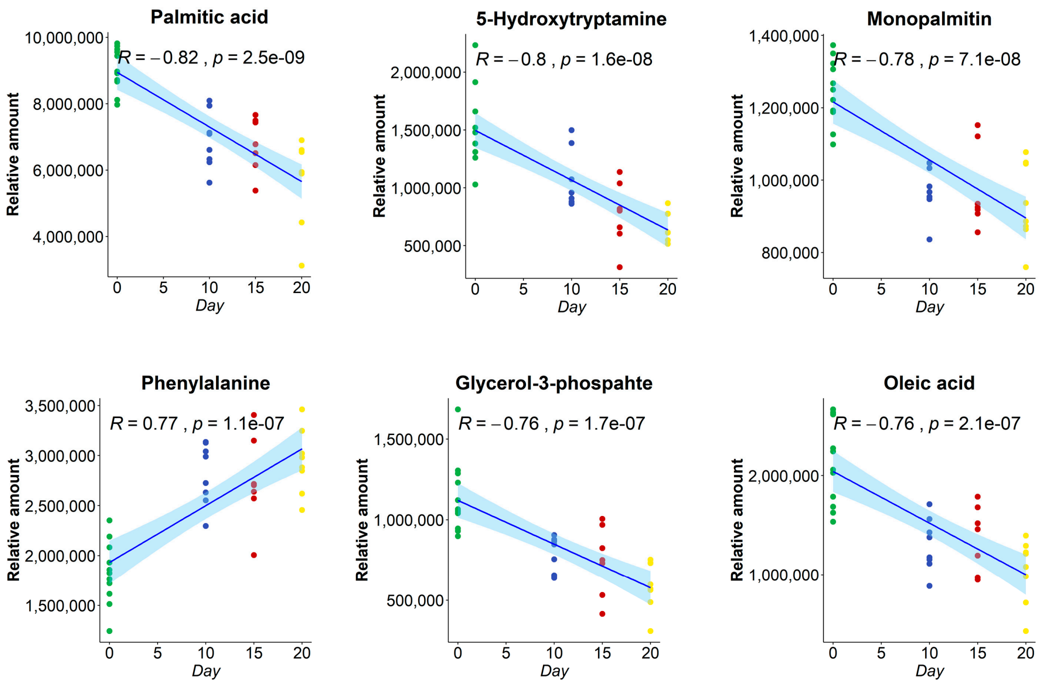

Figure S1 Correlation analysis between metabolites in the serum with drug abuse time.

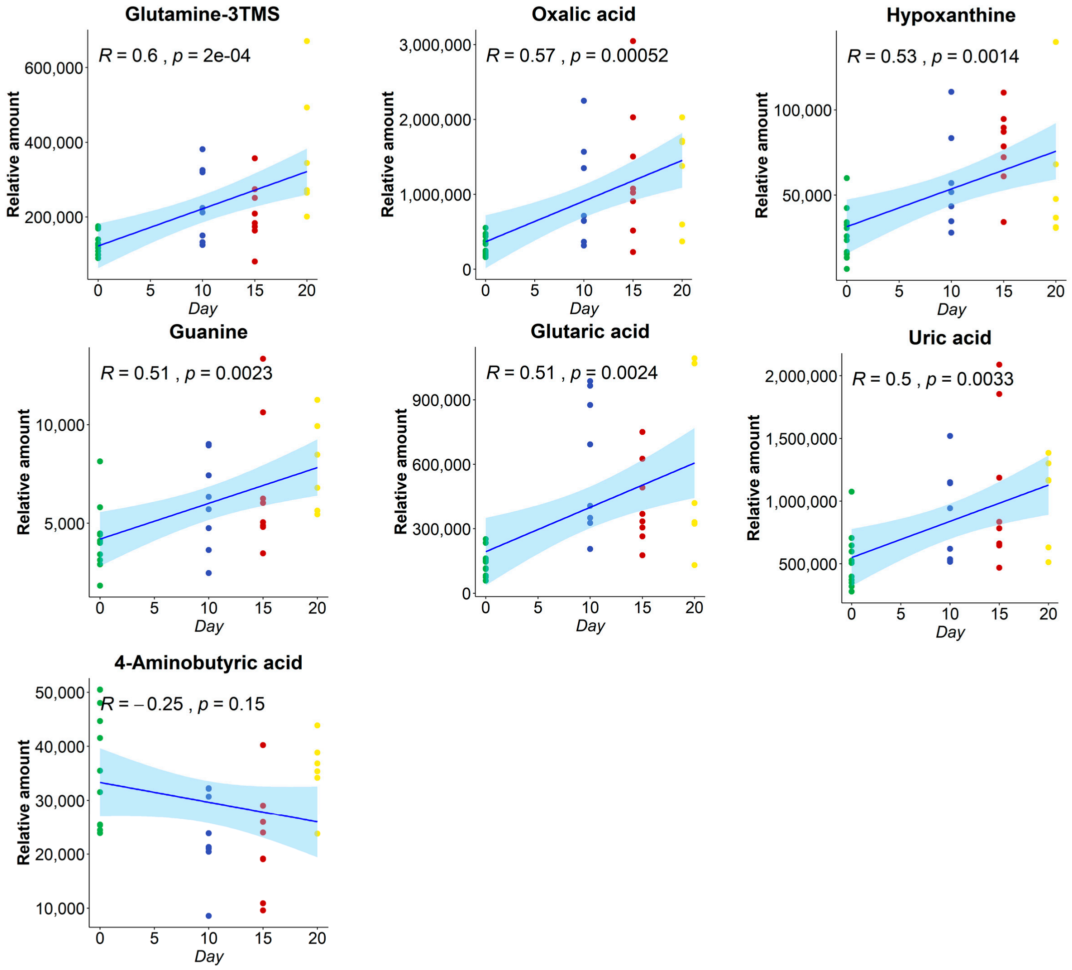

Figure S2 Correlation analysis between metabolites in the urine with drug abuse time.

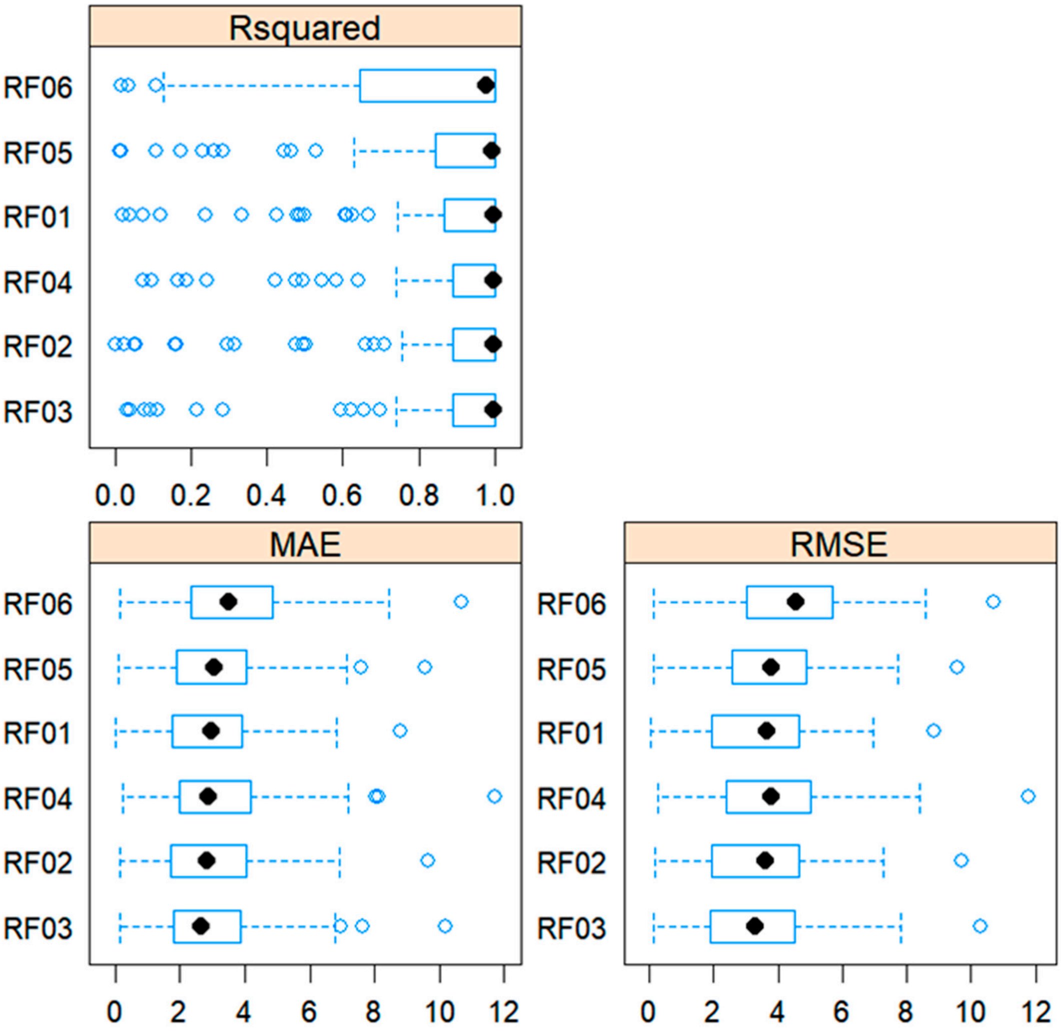

Figure S3 RF model established by different numbers of metabolites in serum. RF01, palmitic acid, 5-hydroxytryptamine, monopalmitin, phenylalanine, glycerol-3-phospahte, oleic acid; RF02, palmitic aceid, 5-hydroxytryptamine, monopalmitin, phenylalanine, glycerol-3-phospahte; RF03, palmitic acid, 5-hydroxytryptamine, monopalmitin, phenylalanine; RF04, palmitic acid, 5-hydroxytryptamine, monopalmitin; RF05, palmitic acid, 5-hydroxytryptamine; RF06, 5-hydroxytryptamine. Palmitic acid, 5-Hydroxytryptamine, Monopalmitin and Phenylalanine had the minimum MAE and RMSE.

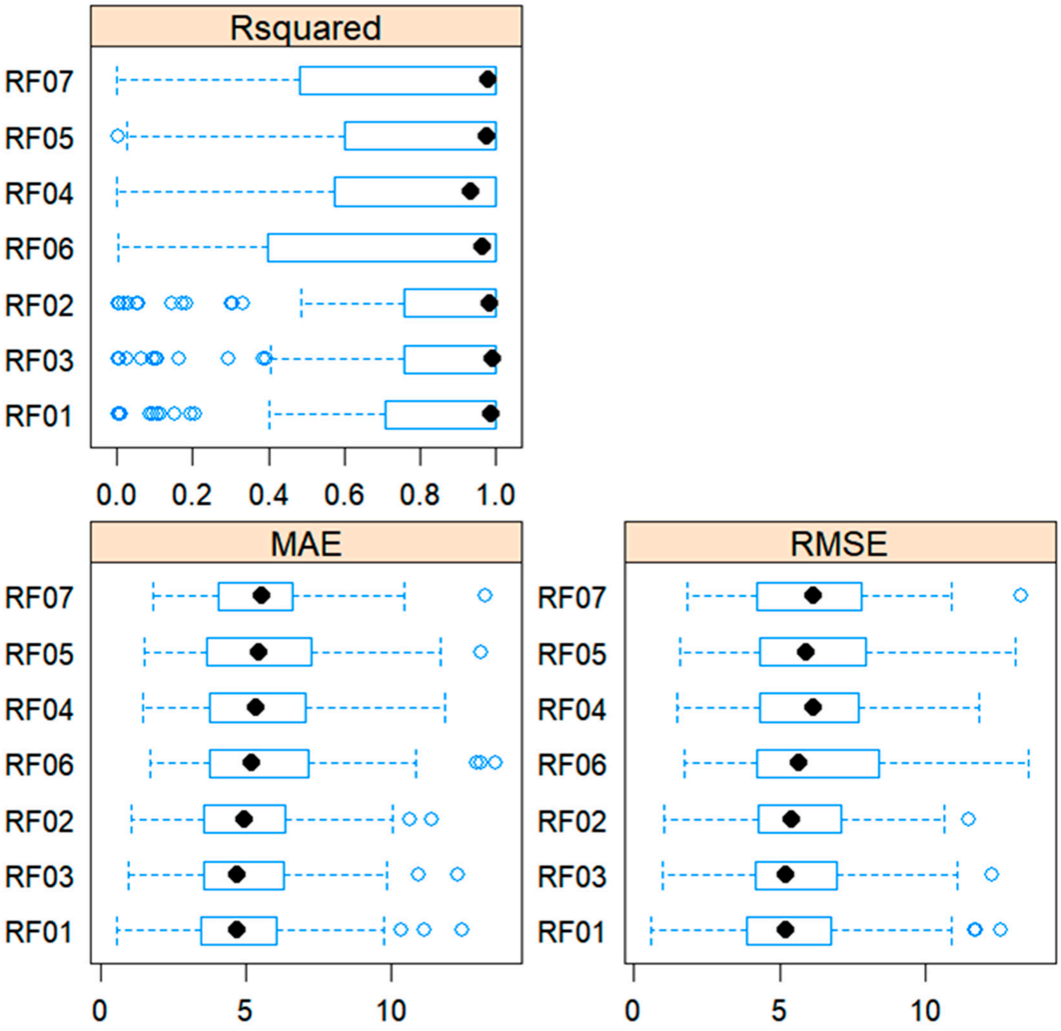

Figure S4 RF model established by different numbers of metabolites in ruine. RF01, glutamine, oxalic acid, hypoxanthine, guanine, glutaric acid, uric acid, 4-aminobutyric acid; RF02, glutamine, oxalic acid, hypoxanthine, guanine, glutaric acid, uric acid; RF03, glutamine, oxalic acid, hypoxanthine, guanine, glutaric acid; RF04, glutamine, oxalic acid, hypoxanthine, guanine; RF05, glutamine, oxalic acid, hypoxanthine; RF06, glutamine, oxalic acid, RF07, glutamine. Glutamine, oxalic acid, hypoxanthine, guanine, glutaric acid, uric acid, 4-aminobutyric acid had the minimum MAE and RMSE.
