# Supplementary material for: Identification of Biomarkers for Methamphetamine Exposure Time Prediction in Mice Using Metabolomics and Machine Learning Approaches
Source: Metabolites. 2022 Dec 10;12(12):1250. doi: 10.3390/metabo12121250 (PMC9780981; doi:10.3390/metabo12121250)
Supplement: Supplementary file 1 [file metabolites-12-01250-s001.zip › Supplemental tables.pdf]

Supplemental Table S1 The identified endogenous compounds in mice serum

Number	Identification	Rt	m/z
1	3-Hydroxypyridine	4.545	152
2	Hexanoic acid	4.740	207
3	Pyruvate	4.670	174
4	Lactate	4.810	191
5	2-hydroxyisobutyric acid	4.855	131
6	glycolic acid	4.995	177
7	Alanine	5.305	116
8	Glycine-TMS	5.515	102
9	Oxalic acid	5.705	220
10	3-Hydroxyisobutyric acid	5.745	177
11	3-Hydroxybutyric acid	5.900	191
12	Monomethylphosphate	6.055	241
13	Urea-3TMS	6.100	261
14	Acetamide	6.260	216
15	Valine	6.435	218
16	Urea-2TMS	6.675	189
17	Serine-2TMS	6.800	6.69
18	Leucine	6.915	158
19	Phosphate	6.915	314
20	glycerol-3TMS	6.920	205
21	Isoleucine	7.100	158
22	Proline	7.150	142
23	Glycine-2TMS	7.215	174
24	Succinate	7.260	247
25	Uracil	7.450	241
26	glyceric acid	7.375	292
27	Fumarate	7.540	245
28	Serine-3TMS	7.605	204
29	Threonine	7.800	218
30	Aspartate-2TMS	8.085	160
31	b-Alanine-3TMS	8.120	248
32	Aminomalonic acid	8.395	218

33	Malate-3TMS	8.500	233
34	Aspartate-3TMS	8.715	232
35	Methionine	8.735	176
36	Pyroglutamate-TMS	8.755	156
37	6-Azauracil	8.840	257
38	Cysteine	8.950	220
39	Creatinine	9.105	329
40	2-Ketoglutaric Acid	9.130	288
41	Glutamine-4TMS	9.230	227
42	Ornithine	9.320	142
43	Glutamate-3TMS	9.335	246
44	Phenylalanine	9.425	218
45	lauric acid	9.505	257
46	Asparagine	9.635	231
47	Taurine	9.660	326
48	Mannonic acid	9.790	275
49	ES	10.005	199
50	Glycerol-3-phosphate	10.130	357
51	Glutamine-4TMS	10.230	227
52	Glutamine-3TMS	10.230	156
53	Hypoxanthine	10.445	265
54	Citrate	10.460	273
55	Ornithine	10.480	142
56	IS	10.660	287
57	Myristic Acid	10.665	285
58	2-Deoxygalactose	10.732	217
59	Fructose	10.785	307
60	Robitol	10.880	319
61	Glucose-1	10.990	319
62	Lysine	11.030	317
63	Tyrosine	11.120	218
64	Pantothenic acid	11.375	420
65	Beta-Methylglucopyranoside	11.52	204
66	Cis-9-Hexadecenoic Acid	11.57	311

67	Xanthine	11.500	353
68	Palmitic acid	11.655	313
69	Uric acid	11.870	441
70	Myo-Inositol	11.875	318
71	Linoleic acid	12.440	337
72	Oleic acid	12.470	339
73	Octadecanoic acid	12.570	341
74	Arachidonic acid	12.575	117
75	Glucose-6-Phosphate	12.860	387
76	Arachidonic acid	13.140	117
77	11-Elcosaenoic acid	13.325	367
78	Myo-Inositol-2-phosphate	13.295	318
79	5-Hydroxytryptamine	13.51	174
80	cis-4,7,10,13,16,19-Docosahexaenoic acid	13.980	117
81	Monopalmitin	13.985	371
82	1-Monostearin	14.810	399
83	Cholesterol-TMS	17.315	458

Supplemental Table S2 The identified endogenous compounds in mice urine

	Identification	Rt	m/z
1	3-Hydroxypyridine	4.550	152
2	Pyruvate	4.670	174
3	Lactate	4.805	191
4	2-hydroxyisobutyric acid	5.000	131
5	glycolic acid	5.000	177
6	Alanine	5.310	116
7	Glycine-TMS	5.505	102
8	Oxalic acid	5.705	220
10	3-Hydroxybutyric acid	5.905	191
9	3-Hydroxyisobutyric acid	5.910	177
11	b-Alanine-TMS	6.220	176
12	2-keto-3-methyl valeric acid	6.270	200
13	Valine	6.44	218
14	Urea-2TMS	6.685	189
15	Serine-2TMS	6.810	116
16	Phosphate	6.910	314
17	Proline	7.185	142
19	Succinate	7.260	247
18	Glycine-2TMS	7.295	174
20	Glyceric acid-3TMS	7.375	292
21	Uracil	7.450	241
22	glyceric acid	7.54	292
23	Fumarate	7.545	245
24	Serine-3TMS	7.585	204
25	Threonine	7.795	218
26	Glutaric acid	7.945	261
27	b-Alanine-3TMS	8.12	248
28	Niacinamide	8.435	179
30	Malate-3TMS	8.495	233
29	Glycine	8.525	216
31	N-Crotonylglycine	8.680	156
32	Methionine	8.735	176

33	Pyroglutamate-TMS	8.755	156
34	γ-Aminobutyric acid	8.805	304
35	Threonic acid	8.840	292
36	Cysteine	8.940	220
37	Creatinine	8.970	115
38	2-Ketoglutaric Acid	9.05	288
39	2-Hydroxyglutaric Acid	9.05	349
40	Glutamate-3TMS	9.330	246
41	N-Acetylaspartate-2TMS	9.555	158
42	Taurine	9.655	326
43	Lyxose	9.820	307
44	ES	9.995	199
45	Aconitic Acid	10.06	375
46	O-ophosphorylethanolamine	10.120	315
47	Glycerol-2-phosphate	10.120	299
48	Glutamine-3TMS	10.265	156
49	Citrate	10.450	273
52	Ornithine	10.505	142
50	Hypoxanthine	10.530	265
53	IS	10.650	287
51	Hippuric Acid	10.655	206
54	Fructose	10.860	307
55	Robitol	10.865	319
56	Glucose	10.975	319
57	Lysine	11.070	317
58	Glucitol	11.225	319
59	Cys-Gly	11.47	333
60	Xanthine	11.490	353
61	Palmitic acid	11.645	313
64	Myo-Inositol	11.855	318
62	Guanine	11.865	352
63	Uric acid	11.865	441
65	5-Hydroxyindoleacetic Acid	12.425	290
66	Octadecanoic acid	12.560	341

67	Cystine	12.895	218
68	Arachidonic acid	13.170	117
69	Uridine	13.565	217
70	Sedoheptulose-7-Phosphate	14.045	387
71	Allonic acid	14.360	217
72	lactose	14.440	361

Supplemental Table S3 Six machine learning model results of the training set and test set in mice serum

	Training set			Testing set		
	RMSE	MAE	Rsquare	RMSE	MAE	Rsquare
PLS	2.528	1.922	0.934	3.201	2.566	0.861
PCR	2.682	1.937	0.881	2.41	2.003	0.924
KNN	3.248	2.276	0.843	3.715	2.6	0.806
SVM	0.773	0.768	0.996	3.195	2.678	0.906
BRNN	4.154	3.556	0.946	4.583	3.905	0.852
RF	1.69	1.482	0.981	2.528	1.922	0.934

Supplemental Table S4 The time prediction results of METH abuse in mice serum from six machine learning model

Time/day	PLS	PCR	KNN	SVM	BRNN	RF
0.00	0.37	0.63	0.00	1.70	5.06	1.07
10.00	11.57	10.31	14.00	12.21	11.64	11.82
15.00	16.77	17.17	13.00	12.29	14.00	14.58
20.00	14.04	16.43	13.00	14.06	12.38	14.92

Supplemental Table S5 Six machine learning model results of the training set and test set in mice urine

	Training set			Testing set		
	RMSE	MAE	Rsquare	RMSE	MAE	Rsquare
PLS	4.628	3.703	0.619	4.184	3.41	0.913
PCR	5.241	4.293	0.511	4.767	4.154	0.977
KNN	5.561	4.357	0.498	6.648	5	0.357
SVM	1.508	1.049	0.972	5.197	4.72	0.838
BRNN	0.808	0.626	0.991	9.797	8.149	0.008
RF	2.369	1.926	0.946	5.094	4.504	0.952

Supplemental Table S6 The time prediction results of METH abuse in mice urine from six machine learning model

Time/day	PLS	PCR	KNN	SVM	BRNN	RF
0	5.54	4.42	8.00	4.91	5.26	3.09
10	11.09	9.68	10.00	11.83	11.74	8.50
15	14.98	11.40	12.00	11.85	0.11	8.34
20	13.90	12.40	8.00	11.79	5.59	12.12

Supplemental Table S7 The time prediction results of METH abuse in mice serum from six random forest models

Time/day	RF01	RF02	RF03	RF04	RF05	RF06
0	1.62	1.68	1.51	1.77	2.13	0.03
10	13.07	12.31	12.32	11.61	10.61	15.51
15	15.33	15.58	16.13	15.55	17.70	15.47
20	16.74	16.81	16.75	17.35	17.98	17.40

Supplemental Table S8 The time prediction results of METH abuse in mice urine from six random forest models

Time/day	RF01	RF02	RF03	RF04	RF05	RF06
0	2.50	1.50	3.12	3.08	2.23	2.65
10	11.34	11.52	7.16	6.59	5.36	1.35
15	14.77	15.27	13.60	13.26	13.62	14.41
20	10.23	9.22	12.77	12.76	12.03	11.47
