# Supplementary figures and images for: Identification of Biomarkers for Methamphetamine Exposure Time Prediction in Mice Using Metabolomics and Machine Learning Approaches
Source: Metabolites. 2022 Dec 10;12(12):1250. doi: 10.3390/metabo12121250 (PMC9780981; doi:10.3390/metabo12121250)

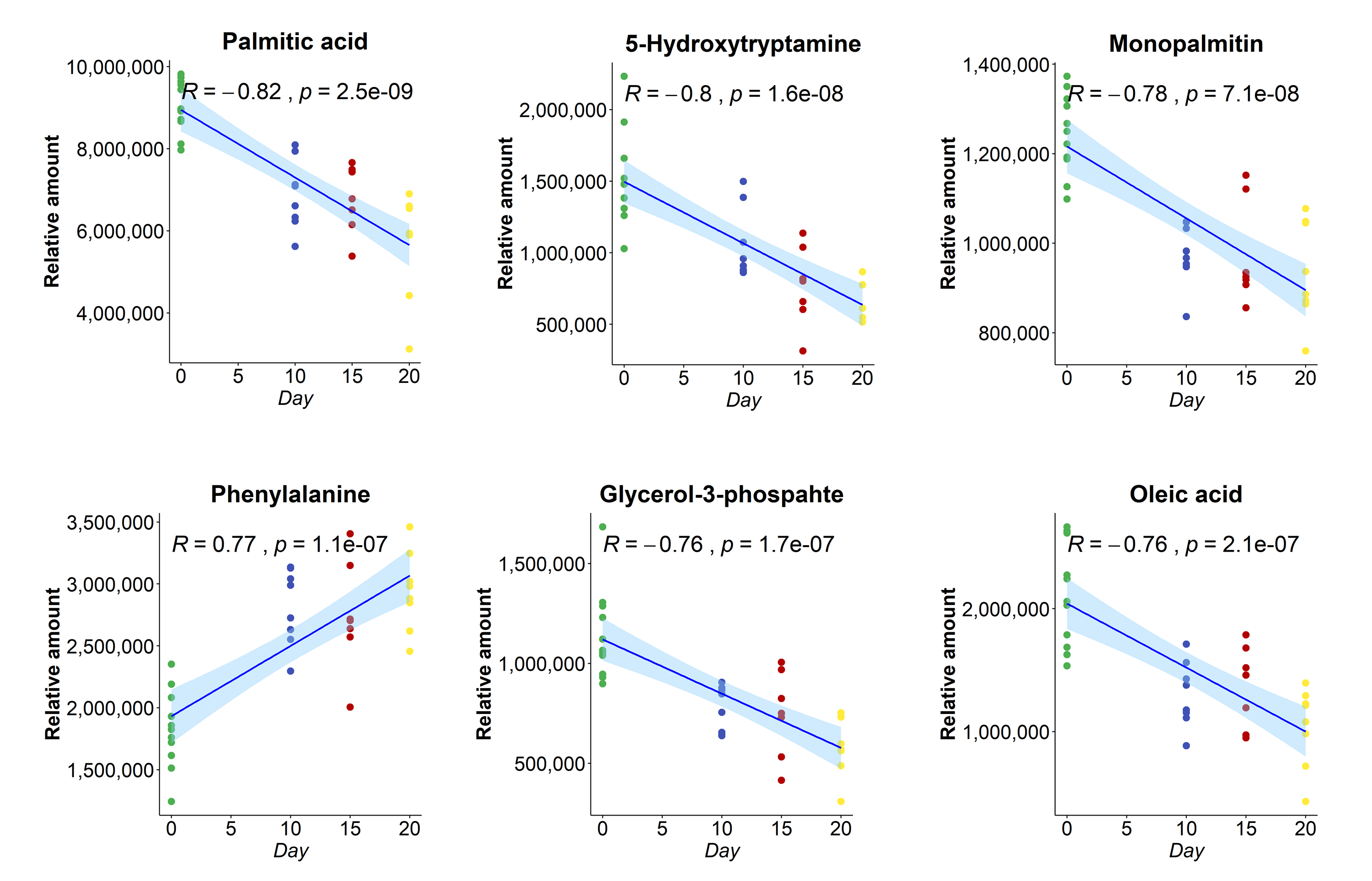

Supplement: Supplementary file 1 [file metabolites-12-01250-s001.zip › Figure S1.tif]

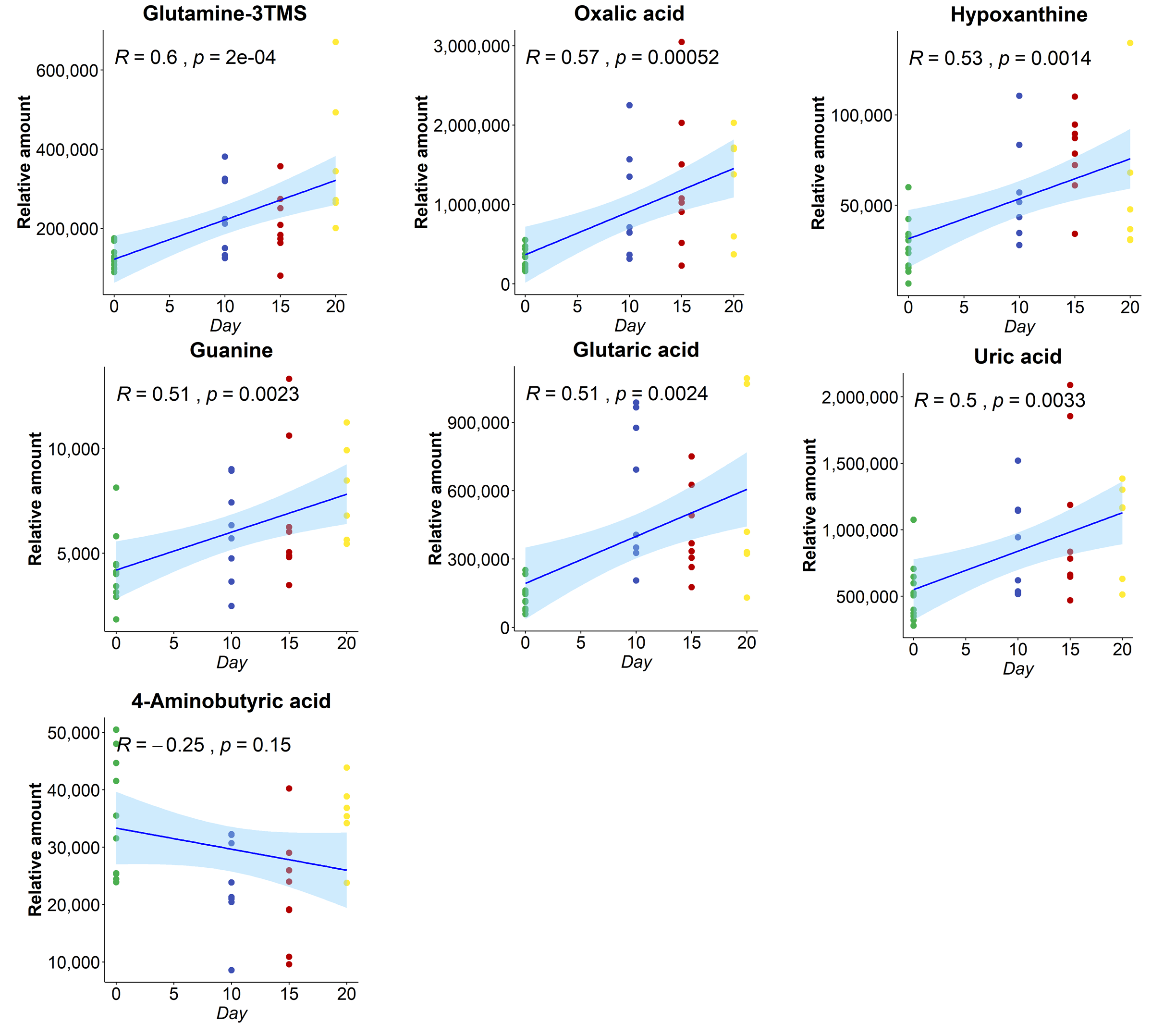

Supplement: Supplementary file 1 [file metabolites-12-01250-s001.zip › Figure S2.tif]

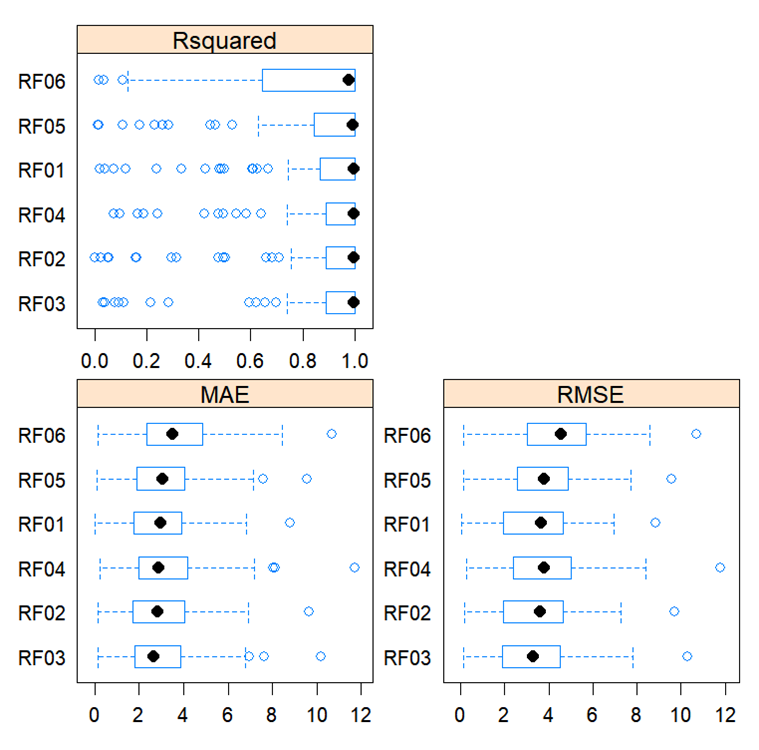

Supplement: Supplementary file 1 [file metabolites-12-01250-s001.zip › Figure S3.tif]

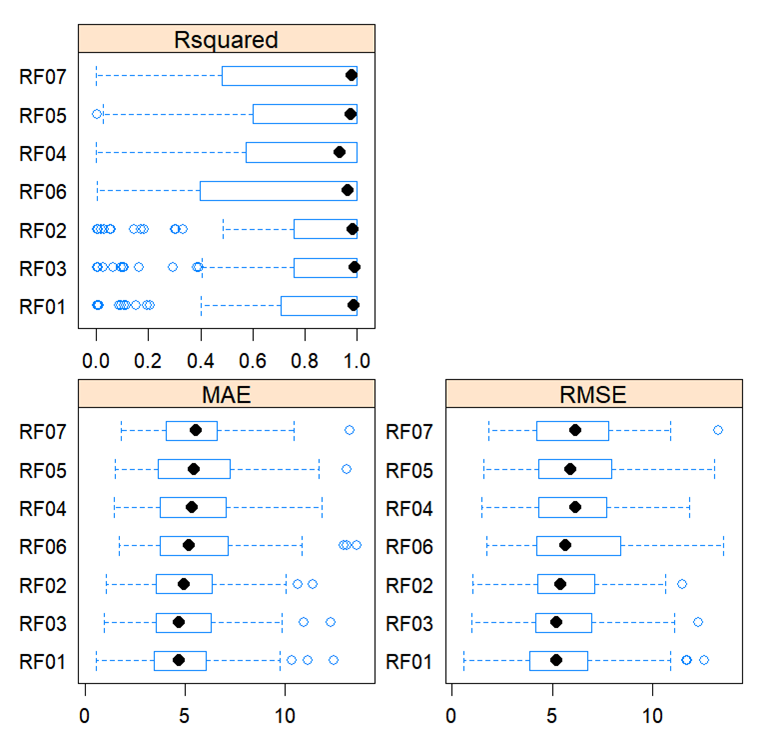

Supplement: Supplementary file 1 [file metabolites-12-01250-s001.zip › Figure S4.tif]
